# REV7 Monomer Is Unable to Participate in Double Strand Break Repair and Translesion Synthesis but Suppresses Mitotic Errors

**DOI:** 10.3390/ijms242115799

**Published:** 2023-10-31

**Authors:** Faye M. Vassel, Daniel J. Laverty, Ke Bian, Cortt G. Piett, Michael T. Hemann, Graham C. Walker, Zachary D. Nagel

**Affiliations:** 1 Department of Biology, Massachusetts Institute of Technology, Cambridge, MA 02139, USA; fvassel@stanford.edu (F.M.V.);; 2 Department of Environmental Health, Harvard TH Chan School of Public Health, Boston, MA 02115, USA

**Keywords:** DNA repair, DNA replication, translesion synthesis, shieldin, cell cycle, FM-HCR

## Abstract

Rev7 is a regulatory protein with roles in translesion synthesis (TLS), double strand break (DSB) repair, replication fork protection, and cell cycle regulation. Rev7 forms a homodimer in vitro using its HORMA (Hop, Rev7, Mad2) domain; however, the functional importance of Rev7 dimerization has been incompletely understood. We analyzed the functional properties of cells expressing either wild-type mouse Rev7 or Rev7^K44A/R124A/A135D^, a mutant that cannot dimerize. The expression of wild-type Rev7, but not the mutant, rescued the sensitivity of Rev7^−/−^ cells to X-rays and several alkylating agents and reversed the olaparib resistance phenotype of Rev7^−/−^ cells. Using a novel fluorescent host-cell reactivation assay, we found that Rev7^K44A/R124A/A135D^ is unable to promote gap-filling TLS opposite an abasic site analog. The Rev7 dimerization interface is also required for shieldin function, as both Rev7^−/−^ cells and Rev7^−/−^ cells expressing Rev7^K44A/R124A/A135D^ exhibit decreased proficiency in rejoining some types of double strand breaks, as well as increased homologous recombination. Interestingly, Rev7^K44A/R124A/A135D^ retains some function in cell cycle regulation, as it maintains an interaction with Ras-related nuclear protein (Ran) and partially rescues the formation of micronuclei. The mutant Rev7 also rescues the G2/M accumulation observed in Rev7^−/−^ cells but does not affect progression through mitosis following nocodazole release. We conclude that while Rev7 dimerization is required for its roles in TLS, DSB repair, and regulation of the anaphase promoting complex, dimerization is at least partially dispensable for promoting mitotic spindle assembly through its interaction with Ran.

## 1. Introduction

Translesion synthesis (TLS), the process by which specialized polymerases (Pols) replicate damaged DNA, is a key pathway that allows cells to tolerate DNA damage. Because many anti-cancer treatments are DNA damaging agents, TLS has recently emerged as a promising target for improving the effectiveness of cancer therapy [1,2]. Inhibitors that target several TLS polymerases or their interactions with other proteins required for their function are being developed [3], including some that take advantage of synthetic lethal relationships between the disruption of TLS polymerase activity and DNA repair defects commonly found in cancers [4]. These findings, together with the importance of TLS polymerases for the mutagenic processes that lead to cancer [5,6], have generated intense interest in the structure and function of TLS polymerases.

Rev7 is a small (23 kDa) protein best known for its role in TLS. Rev7 forms the Pol ζ complex, together with Rev3, the catalytic subunit, and the processivity-enhancing subunits POLD2 and POLD3 [7,8]. Rev7 plays a crucial regulatory role in TLS by tethering another TLS Pol—Rev1—to the Pol ζ complex [9]. Although Rev1 occasionally uses its dCMP transferase activity during TLS, it mainly acts as a scaffolding protein [10,11,12]. The C-terminal domain of Rev1 recruits Y-family Pols such as Pol η or Pol ι to insert a nucleotide opposite a DNA lesion [13]. Subsequently, Pol ζ extends the nascent DNA strand, which often contains a mismatched or minimally paired terminus due to the miscoding potential of many DNA lesions [7,8,14,15]. TLS is therefore understood to involve two Pols in many instances, with one Pol inserting a nucleotide opposite the lesion, and another Pol—frequently Pol ζ—acting as the extender [15,16,17].

Like many other TLS polymerases, Rev7 has emerging roles in numerous pathways. Rev7 has emerged as a critical subunit of shieldin, a multiprotein complex composed of Rev7 and the SHLD1/SHLD2/SHLD3 subunits [18,19]. Shieldin acts downstream of 53BP1 to protect DSB ends from resection, promoting repair by non-homologous end joining (NHEJ), while antagonizing homologous recombination (HR) [19,20,21]. Consequently, loss of shieldin increases HR efficiency in both BRCA1-mutant and BRCA1 wild-type cell lines, rendering them resistant to poly-(ADP)-ribose polymerase (PARP) inhibitors [18,22,23]. Unlike other components of the shieldin complex, REV7 plays a protective role in HR-deficient cells by supporting Pol ζ-dependent filling of single-stranded DNA gaps that accumulate in BRCA-deficient cells [24]. REV7 also provides shieldin-independent protection from resection of stalled replication forks [25], which appears to be distinct from the REV7-independent fork protection pathway carried out by the REV3/SCAI protexin complex [26]. In addition to its roles in TLS, fork protection, and DSB repair, Rev7 plays multiple roles in regulating cell cycle progression. It binds to CDH1, preventing premature activation of the anaphase-promoting complex/cyclosome, which helps ensure faithful progression through mitosis [27]. Rev7 also promotes mitotic spindle assembly and chromosome segregation, possibly via its interaction with Ras-related nuclear protein (RAN) [28,29]. 

Although Cryo-EM structures of the yeast Pol ζ have been reported [12,30], the structure of human Pol ζ and the role Rev7 plays in its assembly have been incompletely understood, in large part due to the difficulty of purifying human Rev3, the largest subunit of the complex [8]. Using a functional complementation assay, Tomida et al. identified a second Rev7 binding motif (RBM) on Rev3, suggesting that Pol ζ may contain two copies of Rev7 [31]. Rev7 homodimerizes in vitro [32], using the canonical HORMA (named after Hop1, Rev7, MAD2 proteins) interface centered around helix αC [33]. The mutation of key residues (K44, R124, and D135) on the Rev7 homodimerization interface causes cellular sensitivity to cisplatin, suggesting that Rev7 homodimerization is required for Pol ζ function [33]. It has also been reported that this triple mutant fails to interact with SHLD2 and is unable to support class-switch recombination or chromosome fusions at deprotected telomeres, consistent with a defect in NHEJ. However, it was unknown how the loss of Rev7 dimerization affects cell cycle regulation and the repair of DSB by other pathways and in other contexts, and the effect of Rev7 dimerization on TLS had not been measured directly in cells, so we sought to directly address these areas. We present data from novel cell-based fluorescence multiplex host-cell reactivation (FM-HCR) assays that reveal the importance of the Rev7 homodimerization interface for the activity of both shieldin and Pol ζ in genome maintenance. We furthermore demonstrate that Rev7 dimerization is at least partially dispensable in promoting chromosome segregation.

## 2. Results

### 2.1. Rev7 Dimerization Is Required for Resistance to DNA-Damaging Agents

**Figure 1:**
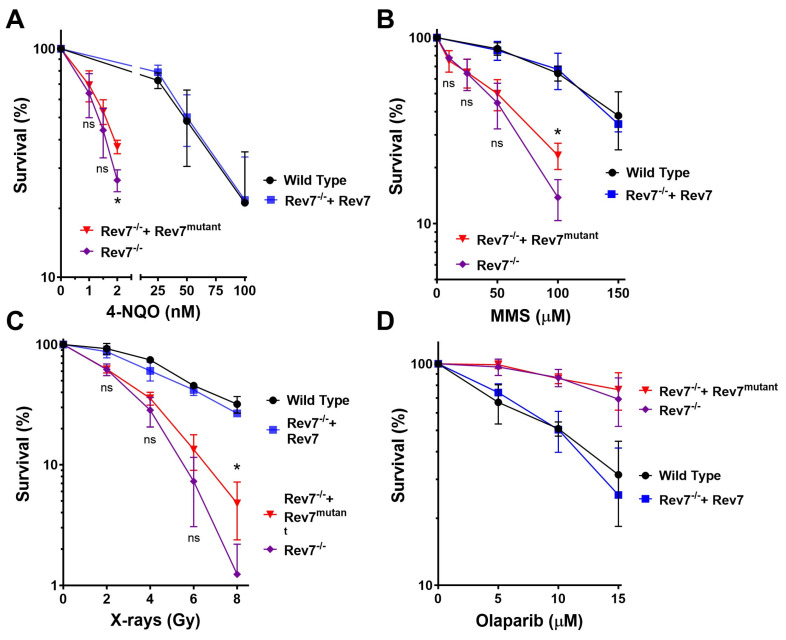
Clonogenic survival of KP cells treated with DNA-damaging agents. Cells were treated with 4-nitroquinolone N-oxide (**A**), methyl methane sulfonate (**B**), X-rays (**C**), or olaparib (**D**). Data are the average of three independent experiments conducted in triplicate. Error bars represent the standard deviation. For statistical analysis, wild type was compared to Rev7^−/−^ +Rev7 and Rev7^−/−^ was compared to Rev7^−/−^ + Rev7^mutant^ using multiple *t*-tests. Wild type vs. Rev7^−/−^ + Rev7: not significant (ns) in all cases. Rev7^−/−^ vs. Rev7^−/−^ + Rev7^mutant^: ns except for the highest dose treatment in 4-NQO (2 nM), MMS (100 µM), and X-rays (8 Gy), which were significantly different, *p* < 0.05, (*). Rev7^mutant^ = Rev7^K44A/R124A/A135D^.

To investigate the functional importance of Rev7 dimerization, we utilized a previously reported Rev7 knockout in the mouse lung cancer KP (Kras^G12D^/Tp53^−/−^) cell line [33,34,35]. Rev7^−/−^ cells were complemented with either wild-type Rev7 or a mutant Rev7 protein (Rev7^K44A/R124A/A135D^, abbreviated Rev7^mutant^) which is incapable of homodimerizing [33]. We confirmed the expression of Rev7 by Western blot and found that Rev7^−/−^ cells complemented with either Rev7 or Rev7^mutant^ expressed high levels of Rev7 protein (Figure S1). To validate our approach, we reproduced the previously reported sensitivity of the Rev7^−/−^ cells to cisplatin and found that clonogenic assays were more sensitive than CellTiter-Glo (Figure S2). We interrogated Pol ζ function by treating with the alkylating agents 4-nitroquinolone N-oxide (4-NQO) and methyl methane sulfonate (MMS), both of which form lesions that are bypassed by Pol ζ [36,37]. As expected, Rev7^−/−^ cells were sensitized to killing by both agents (Figure 1A,B). Complementation with wild-type Rev7 fully reversed the sensitivity of Rev7^−/−^ cells to these agents, while complementation with Rev7^mutant^ had only a minor effect, slightly rescuing cell survival at the highest dose we studied.

We interrogated shieldin function by treating with X-rays, which generate a broad spectrum of DNA lesions, including DSBs. Consistent with impaired DSB repair in cells lacking shieldin. Rev7^−/−^ cells were sensitized to X-rays (Figure 1C). Complementation with wild-type Rev7 reversed this sensitivity, while complementation with Rev7^mutant^ had only a minor effect at the highest dose. We also treated with olaparib, which traps PARP onto DNA, causing replication fork collapse during S phase [38]. Shieldin suppresses end resection, which is required for fork restart, leading to PARP inhibitor lethality [18,21]. Consistent with this phenomenon, knockout of Rev7, an essential component of shieldin, rendered cells less sensitive to olaparib (Figure 1D). Complementation with wild-type Rev7 reversed the olaparib resistance of Rev7^−/−^ cells, while complementation with Rev7^mutant^ did not. Taken together, these results are consistent with the importance of the Rev7 homodimerization interface for TLS and shieldin function but suggest that Rev7^mutant^ may have partial function in other pathways that protect cells from killing with DNA-damaging agents.

### 2.2. The Rev7 Homodimer Suppresses Genomic Instability

**Figure 2:**
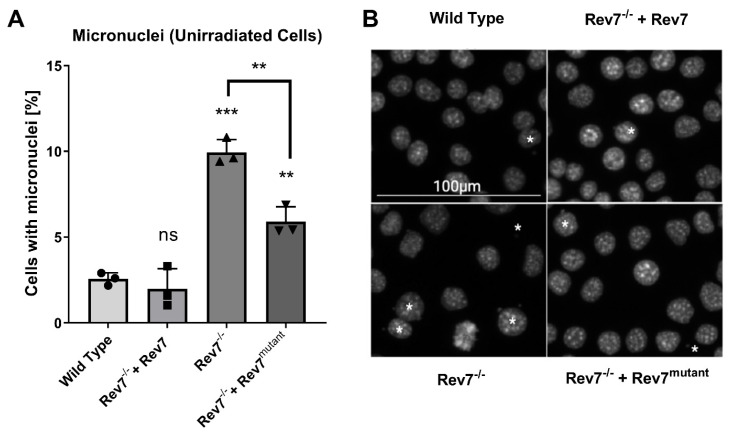
Micronucleus assay for genomic instability. (**A**) Micronucleus assay in KP cells. Each data point shows the average of an independent experiment where 500 cells at identical passage number were counted. Error bars show the 95% confidence interval. Asterisks represent *p*-values from comparison to wild-type cells, unless otherwise indicated (Rev7^−/−^ vs. Rev7^−/−^ + Rev7 ^mutant^). ** indicates *p* < 0.01; *** indicates *p* < 0.001; ns indicates not significant. (**B**) Representative images from A. Asterisks indicate cells with micronuclei, and the asterisk in the top right of Rev7^−/−^ image represents an extracellular micronucleus. Rev7^mutant^ = Rev7^K44A/R124A/A135D^.

We conducted a micronucleus assay in cycling, non-stressed cells to investigate the importance of Rev7 dimerization in the maintenance of genomic stability. A micronucleus is a small extranuclear DNA body formed by misrepair of DSBs, excessive DSBs that overwhelm the repair machinery, or aberrant chromosome segregation during cell division [39]. The knockout of Rev7 led to a greater proportion of cells containing micronuclei, and this phenotype was completely reversed by the expression of wild-type Rev7 (Figure 2). The absolute frequency (~10%) of micronuclei in the Rev7^−/−^ cells is similar to that reported for BRCA-deficient cancer cell lines [40], and the approximately three-fold change comparing WT and Rev7 is larger than the fold-induction reported in most studies comparing cancer patients pre- and post-therapy with DNA-damaging agents [41]. This was consistent with significant substantial genome instability in the Rev7^−/−^ cells and further supported by our analysis of γH2AX foci following treatment with radiation (Figure S3). Interestingly, the expression of Rev7^mutant^ partially rescued the micronuclei phenotype of Rev7^−/−^ cells, further suggesting that the dimerization-defective protein fulfills at least some functions in the cell.

### 2.3. Rev7 Dimerization Is Required for Pol Zeta Function in Gap-Filling TLS

**Figure 3:**
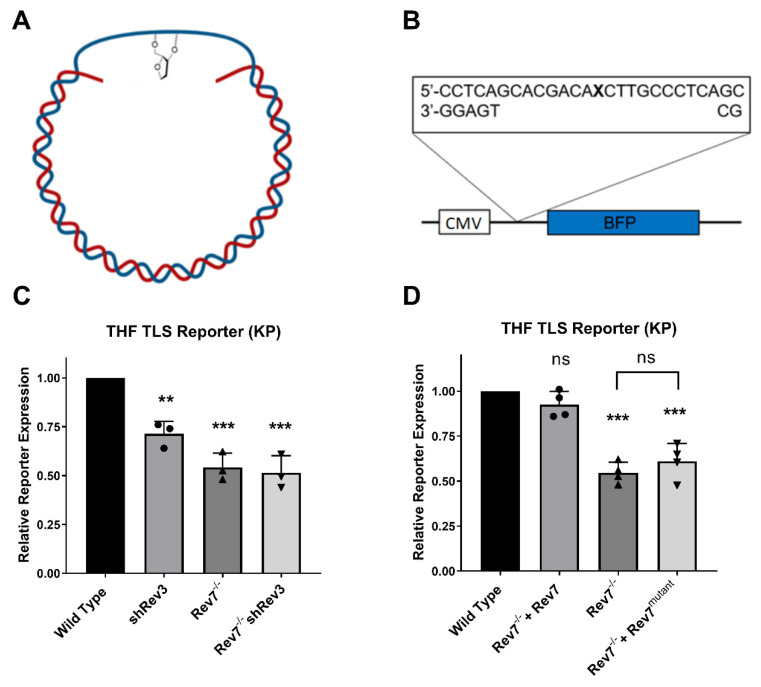
Analysis of gap-filling TLS using a novel fluorescence-based assay. (**A**) TLS reporter assay illustration a site-specific THF lesion in the non-transcribed strand (blue) and a gap in the transcribed strand (red). (**B**) Local sequence of the gapped TLS reporter. X = THF abasic site analog, CMV = cytomegalovirus promoter, BFP = blue fluorescent protein gene. (**C**) Validation of the TLS reporter assay. (**D**) Analysis of the impact of Rev7 dimerization on Pol ζ-mediated TLS. Wild type vs. Rev7^−/−^ + Rev7 *p*-value: 0.088. Rev7^−/−^ vs. Rev7^−/−^ + Rev7^mutant^: *p*: 0.307. Each data point represents an independent experiment and error bars represent the 95% confidence interval. Asterisks represent *p*-values from comparison to wild type by unpaired *t*-test. ** indicates *p* < 0.01, *** indicates *p* < 0.001, ns indicates not statistically significant. Rev7^mutant^ = Rev7^K44A/R124A/A135D^.

The hypersensitivity of cells expressing Rev7^mutant^ to 4-nitroquinolone N-oxide, MMS, and X-rays is suggestive of a defect in TLS. To test this directly, we developed and applied a reporter assay that specifically measures replication through a tetrahydrofuran (THF) lesion, a chemically stable analog of an abasic site (Figure 3A). This assay utilizes gapped plasmids containing site-specific DNA lesions in a manner similar to previously reported assays [16,42,43], but our reporter contains a transcription-blocking gap in the 5′-untranslated region (UTR) of the blue fluorescent protein gene (Figure 3B). Because the gap is in the transcribed strand of the plasmid, fluorescence is restored only when the gap is filled in, which requires replication through THF. To validate that Pol ζ bypasses THF in KP cells, we used shRNA to deplete Rev3 (confirmed by Western blot and RT-qPCR, Figure S4). Consistent with the role of Pol ζ in replicating through THF in KP cells, the depletion of Rev3 significantly decreased TLS reporter expression, as did the knockout of Rev7 (Figure 3C). The depletion of Rev3 in a Rev7^−/−^ background had no additional effect upon TLS efficiency, consistent with the fact that Rev3 and Rev7 are both essential subunits of the Pol ζ complex that carry out TLS. We note that the loss of Rev7 decreased TLS through the THF lesion by approximately 50%, consistent with a similar observation in MEFs following the knockout of Rev3 [16]. Confident that our TLS reporter assay was sensitive to the loss of Pol ζ activity, we assessed the requirement for Rev7 dimerization. The complementation of Rev7^−/−^ cells with wild-type Rev7 restored TLS efficiency to wild-type levels, while complementation with Rev7^mutant^ did not result in a statistically significant change in TLS efficiency (Figure 3D).

### 2.4. Rev7 Dimerization Is Required for Suppression of End Resection by Shieldin

**Figure 4:**
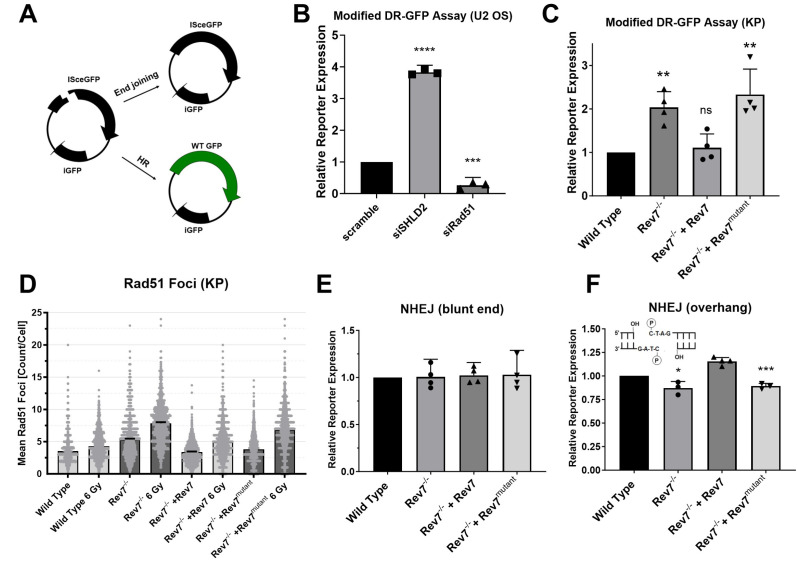
Analysis of double strand break repair capacity using FM-HCR assays. (**A**) Illustration of the modified DR-GFP reporter. (**B**) Validation of DR-GFP assay by siRNA knockdown. (**C**) Measurement of HR efficiency in KP cells using the modified DR-GFP HR reporter assay. (**D**) Rad51 focus assay in KP cells with or without X-ray irradiation (6 Gy). Data are from three independent experiments conducted in triplicate where at least 150 cells were automatically counted in each well. See Figure S5 for representative images. (**E**) End joining reporter assay with blunt ends or (**F**) with compatible overhangs. Error bars show the standard. Asterisks represent *p*-values from comparison to wild type by unpaired *t*-test. * indicates *p* < 0.05, ** indicates *p* < 0.01, *** indicates *p* < 0.001, **** indicates *p* < 0.0001, and ns indicates not statistically significant. Rev7^mutant^ = Rev7^K44A/R124A/A135D^.

The sensitivity of Rev7^−/−^ + Rev7^mutant^ cells to radiation and their resistance to olaparib suggested that Rev7 dimerization is necessary for shieldin activity. Because shieldin suppresses end resection to limit HR, we employed a host-cell reactivation assay to directly measure the importance of Rev7 dimerization for the suppression of HR (Figure 4A). The expression of this reporter plasmid was increased following the depletion of SHLD2 and decreased following the depletion of the HR core factor Rad51 (Figure 4B), confirming that this assay measures DSB repair by HR and is also sensitive to the loss of shieldin function. We found that Rev7^−/−^ cells exhibited greater HR efficiency than wild-type KP cells (Figure 4C). The expression of wild-type Rev7 restored HR efficiency to wild-type levels, whereas the expression of Rev7^mutant^ did not. We further investigated HR efficiency by measuring Rad51 focus formation. Sham-irradiated Rev7^−/−^ and Rev7^mutant^ cells had slightly higher Rad51 foci levels than wild-type cells at baseline (Figure 4D and Figure S5). X-ray irradiation (6 Gy) magnified this difference, with Rev7^−/−^ and Rev7^mutant^ cells exhibiting approximately 50% more Rad51 foci 6 h after 6 Gy irradiation than wild-type and Rev7-complemented cells (Figure 4D), indicating that Rev7 dimerization is necessary for its role in antagonizing HR. These data are also consistent with recent reports of a role for Rev7 in counteracting DNA end resection [25].

We next used a host-cell reactivation assay that measures how efficiently cells rejoin a blunt-ended DSB present in the 5′-UTR of the pMax blue fluorescent protein (BFP) plasmid [44]. The repair of this DSB is primarily dependent upon canonical, PKcs-dependent NHEJ [44]. Surprisingly, in view of a recent report that Rev7 dimerization is required for end joining at exposed telomeres and during class switch recombination [45], the knockout of Rev7 did not affect rejoining of this DSB (Figure 4E). To test whether the structure of the DNA ends influences the requirement for Rev7 dimerization, we generated an additional end joining reporter containing a compatible overhang. A DSB with compatible overhangs was rejoined slightly less efficiently in Rev7^−/−^ cells than in thewild type (Figure 4F). The expression of Rev7 reversed this defect while the expression of Rev7^mutant^ did not.

### 2.5. Rev7 Homodimerization Is Partially Dispensable in Cell Cycle Regulation

**Figure 5:**
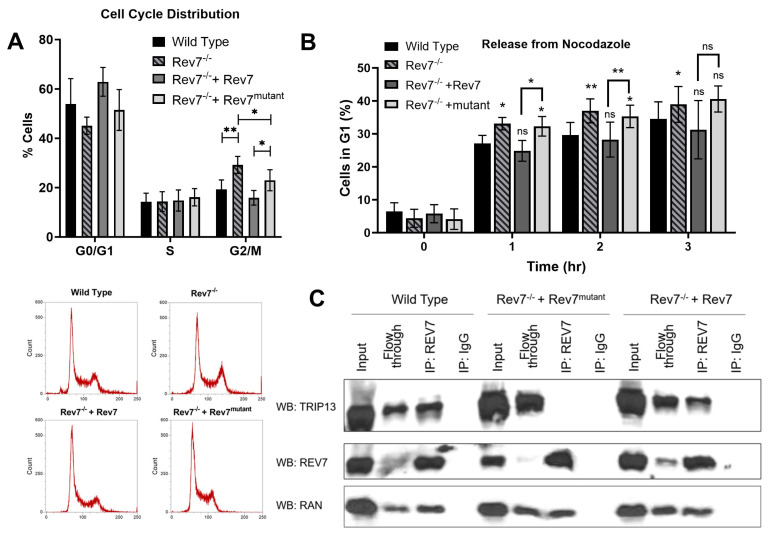
Cell cycle analysis in KP cells. (**A**) Cell cycle distribution of KP cells. Data are the average of five independent experiments, and the error bars show the standard deviation. Wild type vs. Rev7^−/−^ + Rev7^mutant^ *p*-value: 0.169. Representative cell cycle profiles appear below. (**B**) Percentage of cells in G1 phase after release from nocodazole. Data are the average of three independent experiments. (**C**) Co-immunoprecipitation of Trip13 or Ran by anti-Rev7. Protein extracts from the indicated cell line were immunoprecipitated using anti-Rev7 (IP: REV7) or IgG control (IP: IgG control), and eluates along with co-IP input and flow-through were resolved by SDS-PAGE followed by transfer to a PVDF membrane. The membrane was blotted for Ran (WB: RAN), Trip13 (WB: TRIP13), and Rev7 (WB: Rev7). Asterisks represent *p*-values from comparison to wild type by unpaired *t*-test. * indicates *p* < 0.05, ** indicates *p* < 0.01, and ns indicates not statistically significant. Rev7^mutant^ = Rev7^K44A/R124A/A135D^.

We investigated the cell cycle distribution of KP cells and found that Rev7^−/−^ cells showed a slight but significant accumulation in G2/M phase (Figure 5A). The expression of wild-type Rev7 fully reversed G2/M accumulation. Interestingly, the expression of Rev7^mutant^ suppressed G2/M accumulation but to a lesser extent than the expression of wild-type Rev7 in these cells (Figure 5A). We also found that Rev7^−/−^ cells progressed more rapidly through mitosis following nocodazole release (Figure 5B), consistent with a previous report showing that Rev7 prevents premature activation of the anaphase promoting complex/cyclosome [27]. The expression of wild-type Rev7 reversed this phenomenon, while the expression of Rev7^mutant^ did not. 

Our data suggest that Rev7 dimerization is at least partially dispensable for the role of Rev7 in cell cycle regulation. To further investigate this hypothesis, we conducted co-immunoprecipitation experiments to determine whether Rev7^mutant^ interacts with Ran, which was previously proposed as a mediator of Rev7 function in mitotic spindle assembly [28]. We detected a Rev7-Ran interaction in wild-type KP cells and in Rev7^−/−^ cells expressing either wild-type or mutant Rev7, indicating that Rev7 dimerization is not required for this interaction (Figure 5C). We extended our analysis to Trip13, a HORMA domain remodeler which antagonizes Rev7 function in Pol zeta and shieldin. We detected a Rev7-Trip13 interaction in wild-type cells and in Rev7^−/−^ cells complemented with Rev7 (Figure 5), but we could not detect a Rev7–Trip13 interaction in Rev7^−/−^ + Rev7^mutant^ cells, confirming that the Rev7 homodimerization interface is required for the interaction with Trip13, consistent with other reports [45].

## 3. Discussion

Rev7 promotes diverse functions in the cell including TLS, DSB repair, and mitotic spindle assembly. Protein–protein interactions are essential for Rev7’s function in these pathways. For instance, Rev7 forms the shieldin complex with SHLD1, SHLD2, and SHLD3 to antagonize end resection at DSBs [18,19]. Rev7 also forms a multiprotein complex, Pol ζ, with Rev3, POLD2, and POLD3. In the Pol ζ complex, Rev7 mediates the interaction between Rev1 and Rev3, allowing Rev1 to act as a scaffold protein to recruit Y-family Pols that insert a nucleotide opposite the lesion [46]. Pol ζ then extends the nascent strand, taking advantage of its ability to accommodate the mismatched or minimally paired termini often formed by nucleotide insertion opposite a miscoding lesion [7,33,47]. In addition to its functions in DNA repair and damage tolerance, Rev7 regulates mitotic spindle assembly [28]. Consistent with these roles, the knockout of Rev7 sensitizes cells to DNA-damaging agents and causes G2/M arrest [22,28,33]. Spurred by the observation that Rev7 dimerization is necessary for crucial protein–protein interactions in Pol ζ [33], we hypothesized that Rev7 dimerization may also be required for protein–protein interactions in the shieldin complex and with cell cycle regulatory proteins. We expressed either wild-type Rev7 or Rev7^mutant^ in a Rev7 knockout cell line and analyzed DNA repair, genomic instability, and cell cycle progression. We found that KP cells express relatively low levels of Rev7, so the overexpression of Rev7 (using the murine stem cell virus promoter) in Rev7^−/−^ cells led to higher levels of Rev7 than in wild-type KP cells, but importantly, Rev7 levels were similar in Rev7^−/−^ cells complemented with either the wild-type or mutant protein (Figure 1). Furthermore, the overexpression of wild-type Rev7 restored TLS, DSB repair, and drug sensitivity to wild-type levels. 

We found that the Rev7 dimerization interface is important for promoting cellular resistance to diverse DNA-damaging agents including X-rays, 4-NQO, and MMS. Additionally, Rev7^−/−^ cells expressing Rev7^mutant^ are resistant to olaparib, consistent with aberrant shieldin function in these cells [18,22]. Accordingly, our data reveal the disruption of Rev7 dimerization as a potential mechanism of PARP inhibitor resistance. Using fluorescence-based host-cell reactivation assays, we showed that the dimerization interface of Rev7 is required for gap-filling TLS by DNA Pol ζ as well as the suppression of HR. We note that cell cycle differences between the different cell lines (Figure 5A) could potentially affect DSB repair pathway choice, especially HR, which is known to be cell-cycle-regulated [48]. However, since the combination of increased olaparib resistance and decreased NHEJ in Rev7^−/−^ and Rev7^mutant^-complemented cells cannot be attributed to the small cell cycle differences between these cell lines, it is likely that the reporter assays reflect a bona fide increase in HR efficiency.

Interestingly, despite notable phenotypes consistent with defective shieldin activity (increased HR, sensitivity to X-rays, and resistance to olaparib), DSB rejoining efficiency was not diminished in Rev7^−/−^ cells (Figure 4E) when we employed a reporter assay containing a blunt-ended DSB. Conversely, we observed decreased end joining activity for a DSB with an overhangs (Figure 4F). We note that several previous reports used end joining assays where an enzymatically induced DSB contains complementary overhangs. In these reports, consistent with our findings for reporters with overhangs, the depletion of Rev7 or shieldin subunits resulted in decreased NHEJ efficiency [19,49]. The loss of REV7 dimerization also has been reported to disrupt end joining in the context of class switch recombination and at telomeres [45]. Conversely, Feng et al. measured repair by NHEJ, HR, and MMEJ at a DSB formed by CRISPR-Cas9, which mainly forms blunt-end DSBs [50]. Consistent with our findings for a reporter with a blunt DSB, they found that 53BP1^−/−^ MEFs displayed increased usage of HR but no significant decrease in the efficiency of NHEJ [51]. Finally, Schimmel et al. found that theta-mediated end joining is the major mutagenic pathway for repair of blunt-end DSBs, while c-NHEJ is responsible for most mutagenic repair of DSBs with overhang ends [52]. We propose that MMEJ at least partially compensates for defective shieldin function in Rev7^−/−^ cells, especially at DSBs with a blunt end (Figure 4E). Our data also suggest differential requirements for shieldin based on DSB end structure. In particular, REV7 dimerization was required to support its role in the joining DSBs with overhangs (Figure 4E). These data are consistent with recent findings that REV7 protects regressed replication forks, wherein the newly synthesized DNA adopts a structure similar to the DSB in our reporter plasmids that is protected from nucleolytic degradation [25]. Furthermore, our data reinforce recent findings that the Rev7 dimerization interface is required for shieldin function [45].

Intriguingly, the expression of Rev7^mutant^ partially rescued the micronuclei phenotype of Rev7^−/−^ cells even though it failed to rescue TLS or DSB repair. We expect that micronuclei arise in Rev7^−/−^ cells for multiple reasons. Rev7^−/−^ cells are defective in TLS and are therefore expected to accumulate additional strand breaks at endogenously produced DNA lesions due to replication fork collapse and/or nuclease activity at persistent single-stranded gaps. High levels of DSBs, which may be expected in shieldin-defective cells, can also overwhelm cellular repair machinery, leading to misrepair and formation of micronuclei [39]. Recent work indicates that REV3 plays important roles in fork protection, but two recent reports reached opposite conclusions regarding the involvement of REV7 [25,26]. This might reflect the different requirements for stalled replication forks that differ in the structure of the DNA or the DNA lesions present. However, since it was not tested directly in either study, it is tempting to speculate whether some of the fork protection activity of REV3 may be supported by monomeric REV7. Furthermore, Rev7 regulates chromosome segregation during mitosis, so strand breaks may cause micronuclei more frequently in Rev7^−/−^ cells due to defective chromosome segregation. 

The partial reversal of the micronuclei phenotype in Rev7^−/−^ cells complemented with Rev7^mutant^ indicates that the mutant protein promotes some cellular functions. As the expression of Rev7^mutant^ slightly rescues the sensitivity of Rev7^−/−^ cells to multiple DNA-damaging agents (Figure 1) and significantly rescues G2/M accumulation (Figure 5A), we propose that the Rev7 monomer has at least partial function in promoting mitotic spindle assembly. Conversely, the expression of Rev7^mutant^ has no effect on the more rapid progression through mitosis that is observed in Rev7^−/−^ cells released from nocodazole (Figure 5B). This suggests that Rev7 must dimerize to regulate progression through mitosis by suppressing APC/C activation. Although Pol ζ has recently been reported to participate in unscheduled mitotic DNA synthesis (MiDAS) under conditions of replication stress [53], this process did not involve Rev7, making it unlikely that MiDAS can explain the differences in cell cycle progression between Rev7^−/−^ cells and those expressing Rev7^mutant^.

The fact that Rev7^mutant^ only partially suppressed G2/M accumulation suggests two possibilities: (1) Rev7^mutant^ is only partially competent in promoting mitotic spindle assembly or (2) Rev7^mutant^ is fully competent in promoting mitotic spindle assembly, but its inability to promote TLS and DSB repair indirectly causes G2/M accumulation and micronuclei. We favor the second possibility because Rev7^mutant^ was fully proficient for its interaction with Ran in co-IP experiments (Figure 5). 

Recent structural data show that Rev7 dimerization is essential for the assembly of human shieldin and yeast Pol ζ [12,30,54]. Rizzo et al. showed that Rev7 homodimerizes using the canonical HORMA interface centered about helix αC [33]. In the shieldin complex, Rev7 dimerizes using this same interface, with residues such as K44 and R124, which are essential for Rev7 homodimerization, making important polar contacts at the interface of the conformational dimer [54]. However, the Rev7 homodimer in shieldin is also promoted by the SHLD3 subunit, which interacts with both Rev7 monomers and strengthens the interaction between the two. Further investigation is needed to address important questions regarding how Rev7 dimerization may affect its interaction with the shieldin complex and its recruitment to sites of DNA damage. Notably, a recent structure of yeast Pol ζ showed that a Rev7 dimer acts as the organizing center of Pol ζ. In this structure, Rev7 forms a novel head-to-tail dimer, with the αC helices of each monomer quite far apart. Instead, the αC helix (the head) of one Rev7 monomer interacts with a pseudohairpin at the tail of the other Rev7 monomer. Like shieldin, where the SHLD3 subunit strengthens the interaction between both Rev7 monomers, Rev3 makes numerous contacts with one Rev7 monomer, in a manner that stabilizes the Rev7 dimer. Our data show that K44, R124, and D135 are essential for Pol ζ function in TLS and DSBR in mammals. These residues are not conserved between the mouse and yeast proteins, so it is not possible to determine their specific function from the structure of yeast Pol ζ. However, helix αC of one Rev7 monomer lies at the dimerization interface, so it is possible that amino acid substitution at these residues directly disrupts Rev7 dimerization. Our data are broadly consistent with recent structural data showing the importance of Rev7 dimerization for the function of Pol ζ and shieldin and that Rev7 dimerizes in unique ways in different complexes. Future work is needed to determine whether Rev7 dimerization is required for its emerging roles in replication fork protection [25].

We acknowledge several limitations to our work. The KP cell lines derived from the Rev7^−/−^ cell line express higher levels of Rev7 than the parental KP cell line, and the expression levels were not equal between the wild-type- and mutant-complemented cells. Although the level of protein expression could in principle have phenotypic consequences, we note that our results with the Rev7^mutant^ are consistent with previous reports of defects in Pol ζ function for the mutant [33], and when the TRIP13/Rev7 complex is disrupted [22]. Our data also reproduce the finding that Rev7^mutant^ fails to form a complex with TRIP13 and alterations in the ability to support NHEJ in some contexts [45]. Our plasmid-based reporter assays are not expected to reflect the complex structure of chromatin; however, they are highly validated and are uniquely suited to measuring the repair of chemically defined DNA lesions and strand breaks in cells, and they do not require the establishment of a genetically modified cell line. While the plasmid-based reporter assays (Figure 4C) and an analysis of Rad51 foci (Figure 4D) both suggest increased HR in the Rev7^−/−^ and Rev7^mutant^ cells, future work could strengthen these findings using end resection assays [55] or DNA combing assays [56].

In summary, our results are consistent with recent reports on the structure of Pol ζ and shieldin and the importance for Rev7 dimerization in each [12,30], and our findings reinforce those made in a report that appeared recently [45]. Furthermore, our characterization of the DNA repair landscape, DNA damage sensitivity, cell cycle profile, and genomic instability of Rev7^−/−^ cells show that cells expressing a mutant form of Rev7 that cannot dimerize are incompetent for TLS and shieldin function, sensitive to several therapeutically relevant DNA-damaging agents, but partially proficient in cell cycle regulation, most notably promoting chromosome segregation via an interaction with Ran. These findings provide new insights into possible strategies for sensitizing cancers to treatment. Finally, our novel cell-based reporters for TLS and NHEJ provide powerful new FM-HCR assays for assessing the functional consequences of loss or mutation of TLS polymerases in genome maintenance.

## 4. Materials and Methods

### 4.1. Cell Lines

U2OS cells were purchased from ATCC. The Rev7^−/−^ and Rev7-complemented cell lines were generated from the mouse KP cell line (a gift from Tyler Jacks’ lab, Department of Biology, Massachusetts Institute of Technology) and reported previously [33,35]. The previously generated cell line expressing Rev7^mutant^ also co-expressed mCherry, so we generated a Rev7^−/−^ line complemented with wild-type Rev7 and co-expressing mCherry to enable the analysis of both genotypes with the same GFP expressing reporters in FM-HCR experiments. A gBlock gene fragment encoding mouse Rev7 with EcoRI/XhoI restriction sites was subcloned into the pMSCV-IRES-mCherry vector (Addgene) and lentivirally infected into Rev7^−/−^ cells. Cells expressing mCherry were selected by fluorescence-activated cell sorting (FACS), and Rev7 expression was confirmed by Western blot.

KP cells with stable Rev3 knockdown were generated using short hairpin RNA (shRNA). shRNA constructs were designed and cloned as previously described [57]. The vector co-expressed E2Crimson under the control of the SV40 promotor and is identical to the published MSCV/LTRmiR30-SV40-GFP (LMS) vector. Retrovirally infected cells were selected based on E2Crimson expression using FACS and knockdown was confirmed by Western blot and by RT-qPCR using SYBR green in a BioRad thermal cycler with the following primers: REV3L forward: 5′-ACCAGCTCCTCCAGAAGTGA and REV3L reverse: 5′-ATGTCGGTCAAAGGAACCT. 

### 4.2. Cell Culture

KP cells were cultured in DMEM medium supplemented with 10% fetal bovine serum and were maintained below 80% confluence. TK6 cells were cultured in RPMI with 10% fetal bovine serum and maintained below 1 million cells per mL. Mycoplasma testing was conducted at least every three months using the MycoAlert^TM^ mycoplasma testing kit (Lonza, Basel, Switzerland, LT07-318). All cell lines were confirmed to be negative for mycoplasma.

### 4.3. Chemicals

Olaparib was purchased from SelleckChem, while nocodazole, methyl methanesulfonate, and 4-nitroquinolone N-oxide were from Sigma Aldrich (St. Louis, MO, USA).

### 4.4. Enzymes

The enzymes used to generate FM-HCR plasmids were from New England Biolabs (NEB, Ipswich, MA, USA).

### 4.5. Oligonucleotides

Oligonucleotides and gBlocks were ordered from Integrated DNA Technologies. Oligonucleotides used as PCR primers were used without purification, while those used for generating reporter plasmids were purified by denaturing polyacrylamide gel electrophoresis. 

### 4.6. Western Blotting

Cells were lysed with Tris-SDS lysis buffer supplemented with 1× protease inhibitor cocktail (cOmplete EDTA-free, 11873580001, Roche, Basel, Switzerland). The protein concentration of cell lysates was determined by Pierce BCA protein assay (23225, Thermo Fisher Scientific, Waltham, MA, USA). Total protein was separated on 4-20% Mini-Protean-TGX gradient SDS-PAGE gels (BioRad, Hercules, CA, USA) and then transferred to PVDF membranes (IPVH00010, EMD Millipore, Burlington, MA, USA) for blotting.

### 4.7. Antibodies

Anti-Rad51 (rabbit: Abcam, ab133534) 1:1000 dilution IF;

Anti-Rev7 (rabbit: Abcam, ab180579) 1:1000 dilution WB;

Anti-Trip13 (rabbit: Abcam, ab128153) 1:1000 dilution WB;

Anti-beta Actin (Mouse: Abcam, ab49900) 1:10,000 dilution WB;

Anti-DNA Polymerase Zeta antibody (rabbit: Bioorbyt, orb315685) was used at 1:500 dilution (WB) to detect Rev3;

Anti-Ran antibody (mouse: BD Biosciences 610340) 1:1000 dilution WB.

### 4.8. Clonogenic Survival Assays

For assays with 4-NQO, MMS, cisplatin and olaparib, cells were plated at either 125 or 250 cells per well in a 12-well plate in triplicate. The following day, cells were treated with the indicated agent or with vehicle (DMSO). After treatment (24 h for 4-NQO and MMS and 48 h for olaparib), the medium was removed by aspiration, the cells were washed once with PBS, and a new medium was added. For assays using X-rays, cells were irradiated in suspension at 500 cells/mL and were plated in 6-well plates at densities ranging from 100 cells per well to 1000 cells per well. Eight days after plating, cells were fixed with methanol and stained with a solution of 0.5% crystal violet in 25% methanol/75% water. The identity of the cell line was obscured and colonies (at least 50 cells) were manually counted. Survival was calculated by dividing the number of colonies in the treated wells by the colonies in untreated (or vehicle-treated) wells. Representative images of plates stained for colonies appear in Figure S6.

### 4.9. Micronucleus Assay

Cells were plated at 10,000 cells per well in glass-bottom 96-well optical plates (Thermofisher, 164588) in the absence of cytotoxic stress. After 24 h, the medium was removed, and cells were washed with 1X PBS and fixed with 4% paraformaldehyde. Cells were permeabilized with 0.2% Triton-X (VWR, 97062-208) in 1X PBS for 10 min at room temperature. DNA was stained with DAPI (Thermofisher, 62248) at a concentration of 1 ng/mL in 1X PBS, followed by a brief wash with deionized water prior to fixation. Images were acquired with a 20× objective lens using the CellInsight CX5 High-Content Screening (HCS) Platform (Thermofisher) and HCS studio software^TM^ (https://www.fishersci.ca/shop/products/hcs-studio-2-0-cell-analysis-software/sx000041a, accessed on 23 October 2023). Micronuclei were imaged and counted manually by an individual blinded to the identity of the cells. Cells that exhibited at least one micronucleus were scored, and the percentage of cells positive for micronuclei was calculated. Three independent experiments were conducted in triplicate. Anaphases were scored manually from the same images. 

### 4.10. Rad51 and γH2AX Focus Assays

Cells were plated at 10,000 cells per well in glass-bottom 96-well optical plates. The following day, three wells for each cell line were pre-cleared and fixed with methanol (sham-irradiated condition). The plate was then irradiated at a dose of 6 Gy. After 6 h (for Rad51) or 24 h (for γH2AX), three wells for each cell line were pre-cleared and fixed (irradiated condition). The method for pre-clearing and fixing was as follows: wells were washed with 1× PBS and pre-cleared using 0.2% Triton X-100 for 30 s to remove the soluble nuclear protein fraction. Cells were then washed with 1× PBS and fixed with methanol for 20 min followed by permeabilization for 10 min with 0.2% Triton-X 100 in 1× PBS for 10 min at room temperature. Cells were blocked with sterile filtered 2% BSA (staining buffer) in 1× PBS for 1 h prior to Rad51 or γH2AX primary antibody incubation, at a working dilution of 1:1000 in blocking buffer for 1 h at room temperature. Cells were then washed at least 6 times with 0.5% Tween-20 (Sigma-Aldrich, P9416) in 1X PBS for 3 min/wash. Following cell washes, the anti-rabbit secondary antibody (Alexa-fluor 594: Thermofisher, A-11012) was diluted to a working stock of 1:500 in staining buffer and incubated for 30 min at room temperature. Cells were washed with 0.5% tween-20 in 1× PBS prior to DAPI staining (see above). 

Rad51 or γH2AX foci and DAPI signal were imaged and quantified using the CX5 imaging platform DNA Damage Assay bio-module to detect nuclear RAD51 foci within the HCS Studio^TM^ software according to manufacturer recommendations. In brief, primary cell events (individual cells) were identified by DAPI signal intensity, while secondary events (RAD51 foci) were detected based on foci pixel size and intensity. To address variations in cell staining or cell density differences during sample preparation, automated fixed exposure settings were gathered and used across each plate for optimal imaging of each channel across biological replicates. This was accomplished by imaging representative wells for each cell line using the automated bio-module features, which acquired images within a suitable dynamic range for each channel, the acquisition settings were employed while imaging each biological replicate to permit accurate foci detection. To be counted as a Rad51 focus, the average intensity in the Alexa-fluor 594 channel exceeded 1200 with a pixel size greater than 6 pixel^2^. All foci and background signal which did not meet these criteria were excluded from automated foci scoring. Manual quality control checks were implemented after each automated quantitation to ensure foci counts were qualitatively accurate with the digital filter settings (i.e., foci intensity and size) used within the bio-module.

### 4.11. Cell Cycle Profiling

For analysis of the asynchronous cultures, 1 million cells were collected from a culture in log phase growth by trypsinization, quenching with complete medium, and centrifugation (400× *g*, 5 min). For the nocodazole release experiment, 100,000 cells were seeded into each well of a 6-well plate and cultured for approximately 30 h. Cells were then treated with nocodazole (250 nM) for 12 h followed by PBS wash (twice) and medium renewal. At the appropriate time points (0, 1, 2, and 3 h), cells were collected as above and pelleted by centrifugation. Cells were washed once with PBS and then resuspended in 500 µL PBS. The suspension was continuously vortexed while ice-cold 70% ethanol (4.5 mL) was slowly added to the mixture. The samples were stored at −20 °C overnight. The following day, samples were centrifuged (400× *g*, 5 min), and the pellets were washed with PBS. The pellet was then resuspended in PBS containing BSA (1%) RNAse A (0.1 mg/mL), and propidium iodide (0.01 mg/mL) and analyzed by flow cytometry using the YL1 laser on an Attune NxT flow cytometer. 

### 4.12. Co-Immunoprecipitation

Antibodies were pre-cleaned before attaching to magnetic beads using the Pierce Antibody Clean-Up Kit (44600, Thermo Fisher Scientific). A total of 10 μg of antibody were covalently attached to magnetic beads (88828, Pierce Direct Magnetic IP/Co-IP Kit, Thermo Fisher Scientific) and incubated with 1mg of protein for 2 h at room temperature while rotating. Immunoprecipitation (IP) was performed according to the manufacturer’s directions (Pierce Direct Magnetic IP/Co-IP Kit, Thermo Fisher Scientific).

### 4.13. Reporter Plasmid Cocktails

The TLS reporter cocktail contained a transfection control plasmid (pMax_GFP, 4 ng), a gapped reporter plasmid bearing a site-specific THF lesion (4 ng, pMax_BFP backbone), and a carrier plasmid (1000 ng) lacking a mammalian expression cassette. The corresponding undamaged cocktail was identical except a gapped plasmid without a lesion was utilized instead of one bearing the THF lesion. For the HR assay, one plasmid cocktail contained linearized DR-GFP HR reporter plasmid (500 ng), a transfection control plasmid (pMax_BFP, 100 ng), and non-fluorescent carrier plasmid (1000 ng). The undamaged cocktail was identical except the linearized DR-GFP was replaced with the wild-type GFP expressing the pDR-GFP-WT plasmid. For the NHEJ assays, cocktails contained a transfection control plasmid (pMax_GFP, 100 ng), a carrier plasmid (1000 ng), and the NHEJ reporter (100 ng). The undamaged cocktail contained pMax_GFP (100 ng), pMax_BFP (100 ng), and carrier plasmid (1000 ng), and in experiments with overhang NHEJ reporters, pMax_mOrange (100 ng).

### 4.14. Fluorescent Host-Cell Reactivation Assays

KP cells growing in T75 flasks were trypsinized, quenched with complete medium, collected, and plated at 100,000 cells per well in two wells of a 6-well plate. The following day, one well was transfected with the reporter plasmid cocktail and the other well was transfected with the cocktail containing only undamaged plasmids. For KP and U2OS cells, Lipofectamine 3000 was used according to the manufacturer protocol. In brief, each transfection utilized P3000 reagent (4 μL) and Lipofectamine 3000 reagent (3.75 μL) mixed with the plasmid cocktail in serum-free medium (200 μL, Opti-MEM, ThermoFisher). TK6 cells were transfected using a Neon electroporation system (ThermoFisher) according to the manufacturer’s instructions. In brief, cells were collected by centrifugation, washed with PBS, resuspended in the provided R buffer, mixed with plasmid cocktail, and electroporated using default settings (1400 V, 20 ms). Transfected cells were then transferred to a 12-well plate containing complete medium that had been pre-equilibrated in a cell culture incubator for approximately 30 min. Cells were incubated for 24 h after transfection, collected (by trypsinization for KP cells), and analyzed by flow cytometry using an Attune NxT flow cytometer. Gating and compensation were established by transfection of single-color controls, and percent reporter expression was calculated as previously described [44]. 

### 4.15. Incorporation of THF into the Modified Pmax BFP Plasmid

Tandem Nb. Bbv Ci nicking sites were inserted into the 5′-UTR of the transcribed strand of the pMax_BFP plasmid using the following PCR primers: 5′-GCATCGCTAGCGTACCTCAGCACGACAATCTGCCCTCAGCGCCACCATGAGCGAGCTG-3′ and 5′-AGCAGTACTGCTAGCGGTACC-3′. The PCR product was linearized with NheI and circularized by ligation with T4 DNA ligase and then amplified in DH5 alpha *E. coli*. The sequence was confirmed by Sanger sequencing (Genewiz). THF was incorporated into the plasmid using previously described methodology [58]. In brief, single-stranded DNA was prepared by treatment with Nt. BspQI followed by exonuclease III treatment. A 10-fold molar excess of oligonucleotide (containing either a THF lesion or an unmodified thymidine) was annealed to 100 µg of single-stranded DNA by heating and slow cooling. The oligonucleotide sequences were as follows: 5′-pCGTACCTCAGCACGACATTCTGCCCTCAGCGC and 5′-pCGTACCTCAGCACGACAXTCTGCCCTCAGCGC, where p = phosphate and X = the THF abasic site analog. T4 DNA polymerase and T4 DNA ligase were added in a one-pot reaction to extend the primer and then to circularize the plasmid. Agarose gel analysis showed that single-stranded DNA was entirely consumed, leaving approximately 50% closed circular DNA and 50% nicked DNA that had not been ligated. T5 exonuclease was added to digest the remaining nicked DNA; then, proteinase K was added to digest all proteins in solution. Polyethylene glycol precipitation removed peptides, oligonucleotides, and nucleotide triphosphates from the mixture, and then, phenol-chloroform extraction removed residual proteinase K. Precipitated plasmids were dissolved in TE buffer. Overall yield was 28 µg for the THF-containing plasmid and 35 µg for the lesion-free plasmid. Agarose gel analysis confirmed closed circular product with minimal nicked DNA present. 

### 4.16. Generation of Gapped Reporter Plasmids for TLS

The THF-containing plasmid (20 µg) or its corresponding undamaged control plasmid (25 µg) were nicked with 1.5 units of Nb. Bbv Ci per microgram of DNA for 1.5 h at 37 °C in NEB CutSmart buffer. Proteinase K (1 µL) was added, and the mixture was incubated at 50 °C for 30 min. Additionally, 100-fold molar excess of a trap oligonucleotide (5′-GCACGACAATCTGCCCTCA) was added and the mixture was heated to 80 °C for 10 min (to release the oligonucleotide liberated by dual nicking) followed by snap cooling in a room temperature water bath. The released oligonucleotide was fully complementary to the trap oligo but contained two mismatches (or one mismatch and a THF lesion) if it were to re-anneal to the gapped plasmid. Polyethylene glycol precipitation followed by phenol-chloroform extraction and ethanol precipitation was employed as in the above section to give 22 µg of gapped THF plasmid and 28 µg of gapped control plasmid. The plasmids were resuspended in TE and stored at 4 °C.

### 4.17. DSB Rejoining Reporters (NHEJ)

ScaI-linearized BFP was reported previously [44,59]. To generate a DSB with a 4-nt overhang, pMax_mOrange (250 µg) was digested with NheI (400 U) for 1 h at 37 °C in the provided CutSmart reaction buffer, purified by phenol-chloroform extraction and ethanol precipitation, and linearization was confirmed by agarose gel electrophoresis. 

### 4.18. Modified DR-GFP Reporter Assay

The pDRGFP plasmid was purchased from Addgene (plasmid #26475). The pDRGFP reporter does not express GFP unless homology-directed repair of the ISce-induced double strand break restores the wild-type sequence using a truncated iGFP fragment donor sequence that lies outside the transcribed region of the plasmid (Figure 4A). The corresponding wild-type plasmid (pDRGFP-WT) was generated by replacing the non-fluorescent ISceI-GFP gene with the wild-type GFP sequence (confirmed by Sanger sequencing and GFP fluorescence in transfected cells). The pDRGFP plasmid was linearized by treatment with the homing endonuclease I-SceI (NEB) in CutSmart buffer. The plasmid was purified by phenol/chloroform extraction and ethanol precipitation. The expression of this reporter was calculated as previously described for other FM-HCR assays, with pDRGFP-WT serving as the undamaged control [44]. 

The reporter was validated by the knockdown of SHLD2 (FAM35A) (siRNA sequence: 5′-AAGGAGUGGUUCUGAUUAA-3′) [60] or Rad51 (siRNA sequence: 5′-AAGCUGAAGCUAUGUUCGCCA-3′) in U2 OS cells (Figure S7).

### 4.19. CellTiterGlo Viability Assays

To assess cell viability following cisplatin-induced DNA damage, cells were seeded in triplicate (8 × 10^3^) in 96-well plates and treated as indicated with cisplatin. After 48 h treatment, cell viability was assessed using Cell Titer-Glo (Promega) on an Applied Biosystems microplate luminometer following the manufacturer’s protocol.

## Data Availability

The data are contained within the article and Supplementary Information.

## Supplementary Materials

The supporting information can be downloaded at https://www.mdpi.com/article/10.3390/ijms242115799/s1.
