# Supplementary material for: REV7 Monomer Is Unable to Participate in Double Strand Break Repair and Translesion Synthesis but Suppresses Mitotic Errors
Source: Int J Mol Sci. 2023 Oct 31;24(21):15799. doi: 10.3390/ijms242115799 (PMC10649693; doi:10.3390/ijms242115799)
Supplement: Supplementary file 1 [file ijms-24-15799-s001.zip › ijms-2617309-supplementary.pdf]

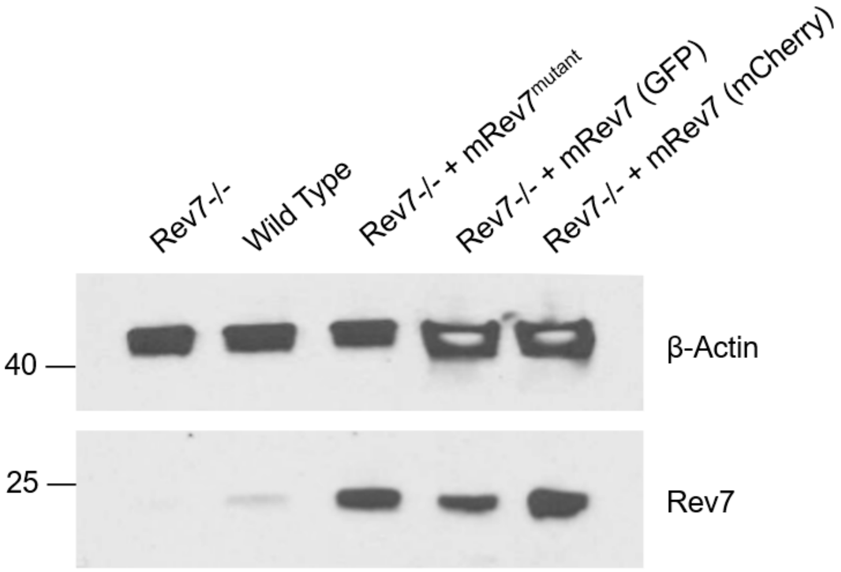

Figure S1. Validation of KP cells used in this study.

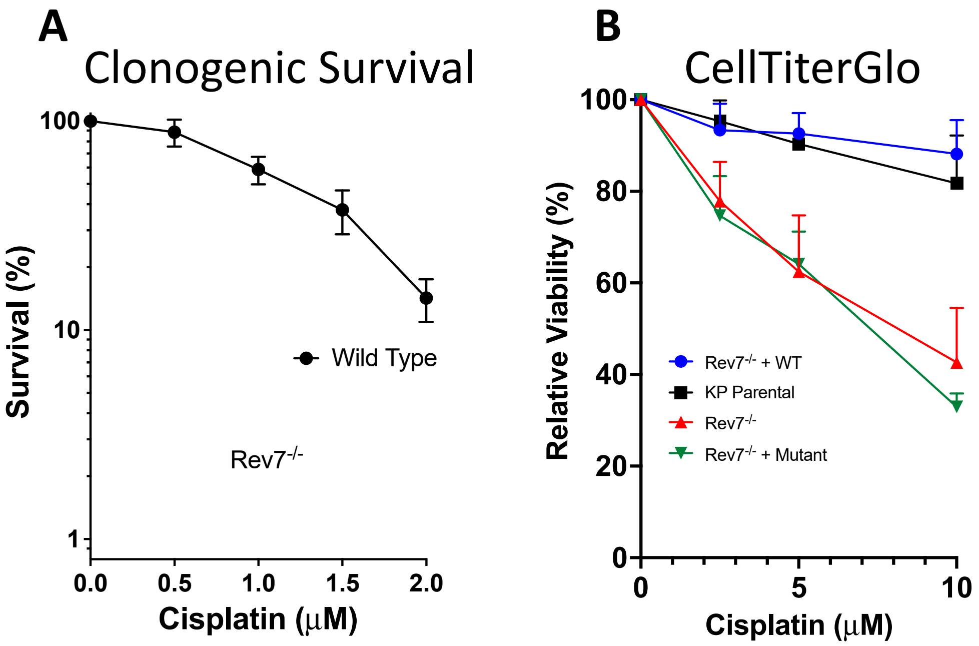

Figure S2. Comparison of (**A**) clonogenic survival or (**B**) CellTiter-Glo viability assay for killing of KP cells with cisplatin.

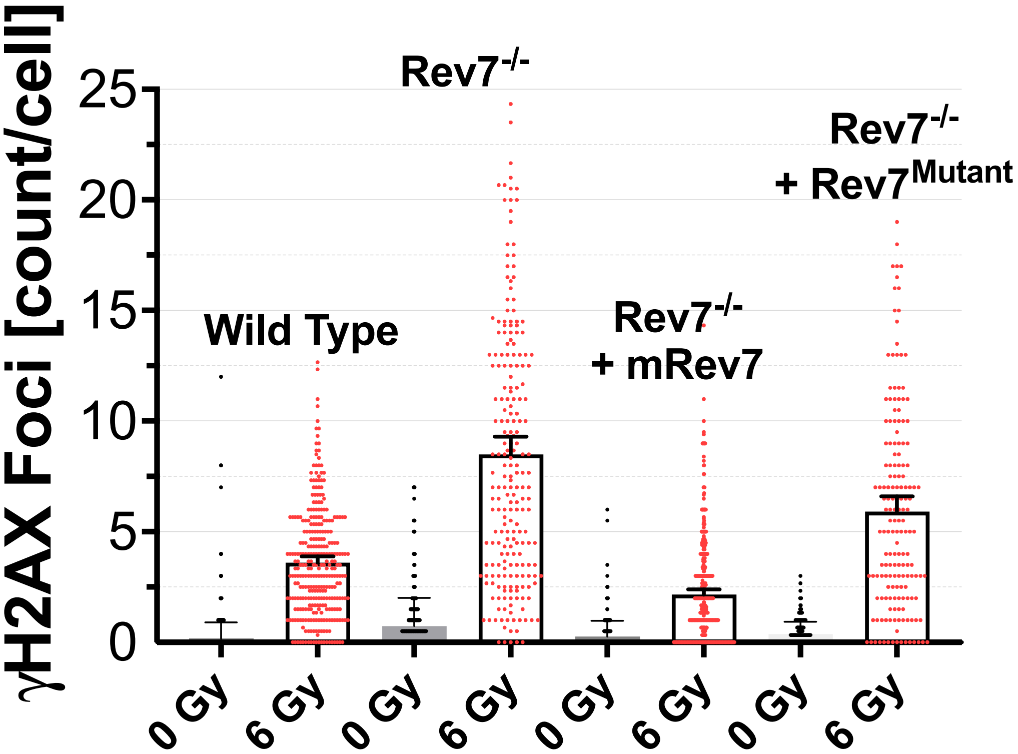

Figure S3. Analysis of gH2AX foci in KP cells that were either mock irradiated (0 Gy) or treated with X-rays (6 Gy). The genotype of the cells is indicated above the columns. Data represent the pooled results of three replicate wells.

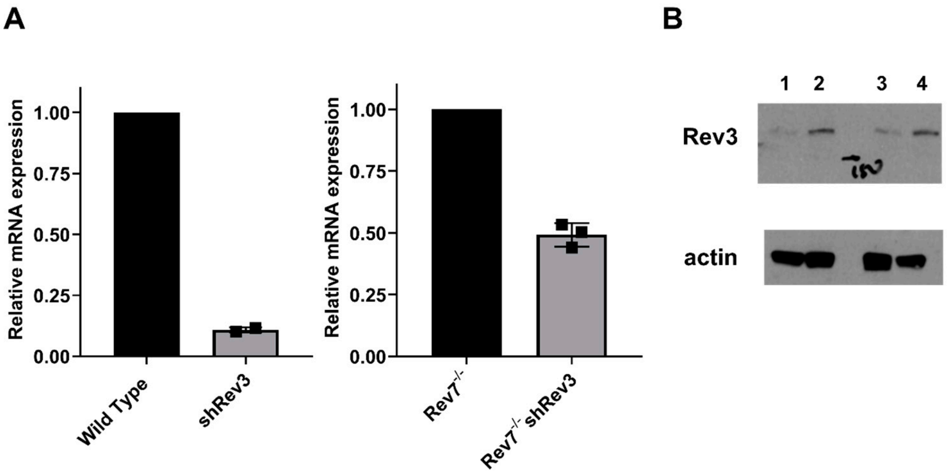

Figure S4. Validation of knockdowns. (**A**) Validation of Rev3 knockdown in KP cells by RT-qPCR. (**B**) Validation of Rev3 knockdown in KP cells by Western blot. 1: shRev3 2: wild type 3: $Rev7^{-/-}$ shRev3 4: $Rev^{-/-}$. The mark between lanes 2 and 3 indicates the 150 kDa molecular weight marker.

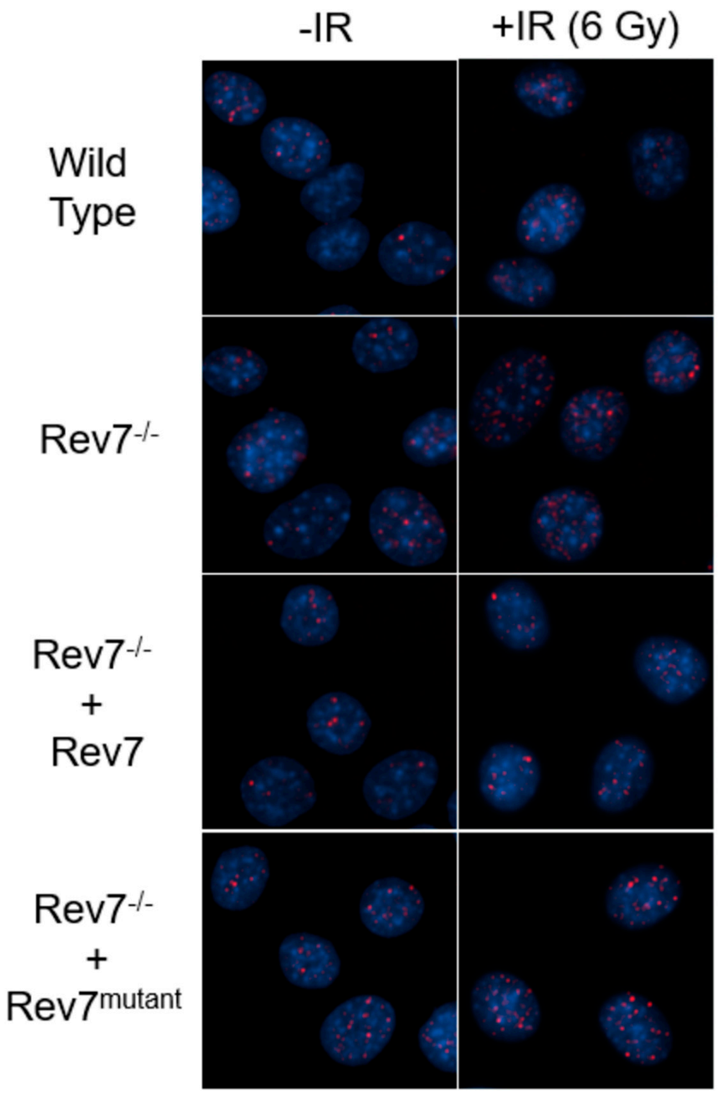

Figure S5. Representative Rad51 focus images (red) of KP cells stained with DAPI (blue).

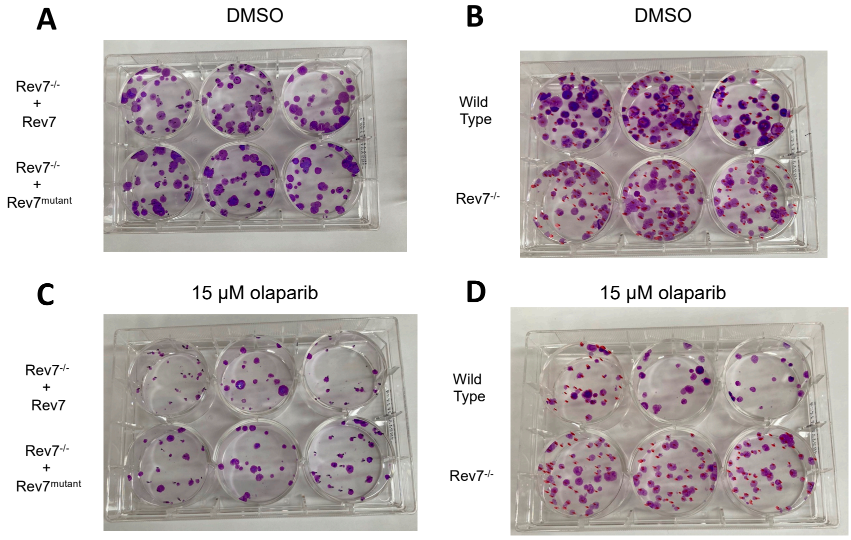

Figure S6. Representative images of crystal violet stained colonies growing in plates from clonogenic survival assay. (**A**) Rev7$^{-/-}$ cells

complemented with wild-type Rev7 (top) or Rev7^{mutant} (bottom) under treatment with DMSO vehicle. (**B**) Parental KP cells (top) or Rev7$^{-/-}$ cells (bottom) under treatment with DMSO vehicle. (**C**) Rev7$^{-/-}$ cells complemented with wild-type Rev7 (top) or Rev7^{mutant} (bottom) under treatment with 15 μM olaparib. (**D**) Parental KP cells (top) or Rev7$^{-/-}$ cells (bottom) under treatment with μM olaparib.

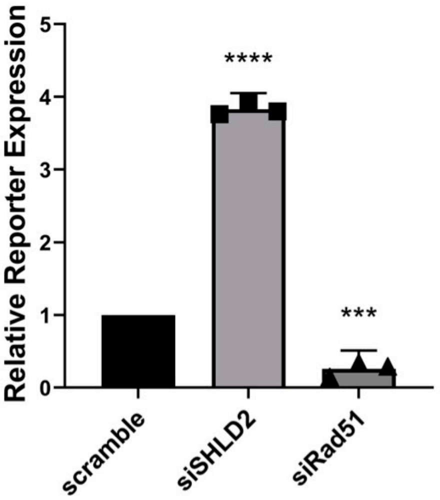

Figure S7. Validation of modified DR-GFP assay. U2OS cells transfected with non-targeting control siRNA (scramble) or siRNA targeting SHLD2 or Rad51 were subsequently analyzed using a functional assay for homology-directed repair. Green fluorescent reporter protein expression in cells transfected with siRNA targeting SHLD2 or Rad51 is reported relative to that of the non-targeting control. Each data point represents an independent experiment; error bars represent the standard deviation of the mean. Statistical significance calculated from an unpaired t-test is represented by **** ($p < 0.001$) or *** ($p < 0.01$).
